# Design of Small MEMS Microphone Array Systems for Direction Finding of Outdoors Moving Vehicles

**DOI:** 10.3390/s140304384

**Published:** 2014-03-05

**Authors:** Xin Zhang, Jingchang Huang, Enliang Song, Huawei Liu, Baoqing Li, Xiaobing Yuan

**Affiliations:** 1. Shanghai Institute of Microsystem and Information Technology, Chinese Academy of Sciences, Shanghai 200050, China; E-Mails: zxinscholar@gmail.com (X.Z.); jchhuang@mail.ustc.edu.cn (J.H.); liuhuawei2008@yahoo.cn (H.L.); libq@mail.sim.ac.cn (B.L.); yuanxb@mail.sim.ac.cn (X.Y.); 2. Science and Technology on Micro-System Laboratory, Chinese Academy of Sciences, Shanghai 200050, China; 3. Graduate University of the Chinese Academy of Sciences, Beijing 100049, China

**Keywords:** direction finding, MEMS microphone array, small aperture arrays, system design, UGS

## Abstract

In this paper, a MEMS microphone array system scheme is proposed which implements real-time direction of arrival (DOA) estimation for moving vehicles. Wind noise is the primary source of unwanted noise on microphones outdoors. A multiple signal classification (MUSIC) algorithm is used in this paper for direction finding associated with spatial coherence to discriminate between the wind noise and the acoustic signals of a vehicle. The method is implemented in a SHARC DSP processor and the real-time estimated DOA is uploaded through Bluetooth or a UART module. Experimental results in different places show the validity of the system and the deviation is no bigger than 6° in the presence of wind noise.

## 1. Introduction

Direction finding of moving vehicles by microphone arrays is very important in unattended ground sensor (UGS) systems [1,2] and intelligent transportation system (ITS) [3]. The ITS is used in the city while the UGS system is used in the battlefield. UGS generally consists of seismic, acoustic, passive infrared and daylight imager sensors. These are small, robust, ground-based intelligence surveillance and reconnaissance (ISR) networked devices that provide an early warning system capable of remote operation under all weather conditions. UGS will detect, track, classify and identify vehicles within their area of operation and report in near real-time.

The bearing of vehicle is an essential piece of intelligence and could also provide assisting information for other sensors. Direction finding is the basis of vehicle detection [4], vehicle counting [5], vehicle tracking [1] and moving vehicle velocity estimation [6]. Furthermore, using the estimated direction, multiple microphone arrays distributed over a planar region could work out the accurate position of vehicle [7–9].

To design a real-time direction finding system, it is very important to choose a suitable DOA estimation method. The criteria for choosing the method are given below:

- Low complexity for real-time processing
- High accuracy for the performance of the system
- Moderate sampling rate for the hardware load

In general, methods for acoustic source direction finding can be divided into three categories based on their increasing computational complexity: time-delay-based methods [10–12], spectral-based methods and parametric methods [13,14]. In time-delay-based methods the Time-Difference-Of-Arrival (TDOA) is obtained from the phase differences of microphones [15], and the performance of time delay estimation is dependent on the sampling rate. When the array aperture is small, time-delay-based methods have a high sampling rate, which worsens the load on the hardware system [16]. Parametric methods feature high computational cost [17] and thus are not suitable for real-time processing, while spectral-based methods such as the MUSIC [18], Root-MUSIC [19] and ESPRIT algorithms [20] are computationally attractive, while providing high accuracy. In addition, the performance of spectral-based methods is independent of the sampling rate as long as the Nyquist-Shannon sampling law is satisfied.

Another challenge for a microphone array in the field is the wind noise. In this paper we propose a spatial coherence-based method to estimate the useful band for vehicle direction finding. The sound of the vehicle in the field has free field characteristics and the wind noise has the characteristics of a noise field. According to reference [21], spatial coherence could be used to distinguish between the noise of the wind and the sound of the vehicle for each frequency bin.

In this paper, we design and implement a vehicle direction finding system using four MEMS microphones, a SHARC DSP processor, MAXIM simultaneous-sampling ADCs and supplemental hardware circuits. The real-time estimated DOA could be reported through a Bluetooth or UART module. The interference of wind noise in the field is reduced through estimation of the useful frequency band by spatial coherence. Because the designed aperture of the array is small and the acoustic signal of the vehicle is band limited, we use the MUSIC algorithm for its relatively low complexity and high accuracy.

The remainder of this paper is organized as follows: Section 2 presents the hardware design of the microphone array. Section 3 elaborates the signal processing method and software design. It illustrates the direction finding method and the solution to wind noise. System verification and experimental results with MEMS microphone array are given in Section 4 and conclusions are presented in Section 5.

## 2. Hardware Design

In this section, we first elaborate our choice of the microphone array geometry, and then describe the design of system architecture.

### 2.1. Microphone Array Geometry

The number of microphones in the array and the array aperture are determined by the following requirements:

- The array must have the same resolution in all directions
- The vehicle signal occupies the frequency band from 100 Hz to 3,000 Hz [22]. The aperture of the array has to satisfy the spatial sampling criterion in the entire frequency band to avoid performance degradation due to spatial aliasing
- The microphone array system should achieve high accuracy

In general, uniform circular arrays have the same resolution in all directions and the uniform array could provide enough space for circuit design. Furthermore, to satisfy the spatial sampling criterion *d* ≤ 0.5λ, the array aperture should be no bigger than 5 cm, where *d* is the minimum distance between any two array microphones, and λ is the wavelength of the acoustic signal.

**Figure 1.**
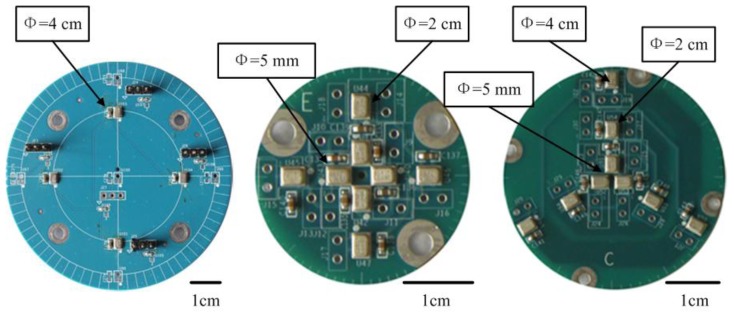
Microphone arrays designed for verification of accuracy of direction finding. The MEMS microphone is ADMP504. *Φ* is the aperture of the array.

To simplify the complexity of the system design, we decided to use no more than four microphones. The expected accuracy of the direction finding system is less than 6°. To determine the number of microphones and the aperture of the array, different microphone arrays were designed (Figure 1). Simulation and experimental results are shown in Table 1.

The 10 dB and 20 dB level experiments are conducted by 500 Monte Carlo simulations. The room experiments are conducted using the microphone arrays shown in Figure 1 by putting them on a turntable. The acoustic source is Jasmine (Molihua) a famous Chinese folk song played by a piano that is fixed to 0°.The array turns around on the turntable at a constant rotation speed of 25.7°/s. As shown in Table 1, even though the simulations show that both three and four microphone arrays with aperture of 4 cm have desirable accuracy, based on the room turntable experiments, we decided to choose the 4 cm uniform circular array with four microphones.

### 2.2. System Architecture

**Figure 2.**
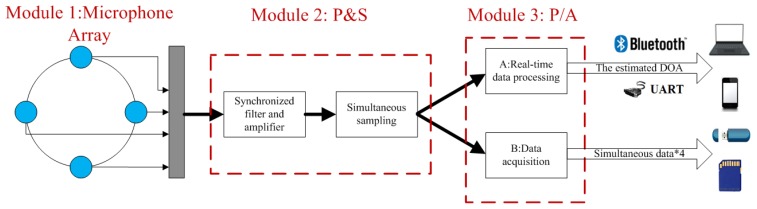
Block diagram of the MEMS microphone array system.

The block diagram of the prototype MEMS microphone array system is depicted in Figure 2. The system is divided into three modules by function: microphone array (Module 1), preprocessing and sampling module (Module 2: P&S) and real-time processing or data acquisition module (Module 3: P/A). The microphone array is a 4 cm uniform circular array with four MEMS microphones, after preprocessing of synchronized filters and amplifiers, simultaneous sampling ADCs are used to capture signals from the microphones. The synchronized filters and amplifiers mean that a strict demand on the consistency of the four channels is requested. The function of module P/A is configured by users, either for real-time processing by a DSP using the proposed method or to store the signals in the memory device through a data acquisition interface for appropriate posterior analysis.

**Figure 3.**
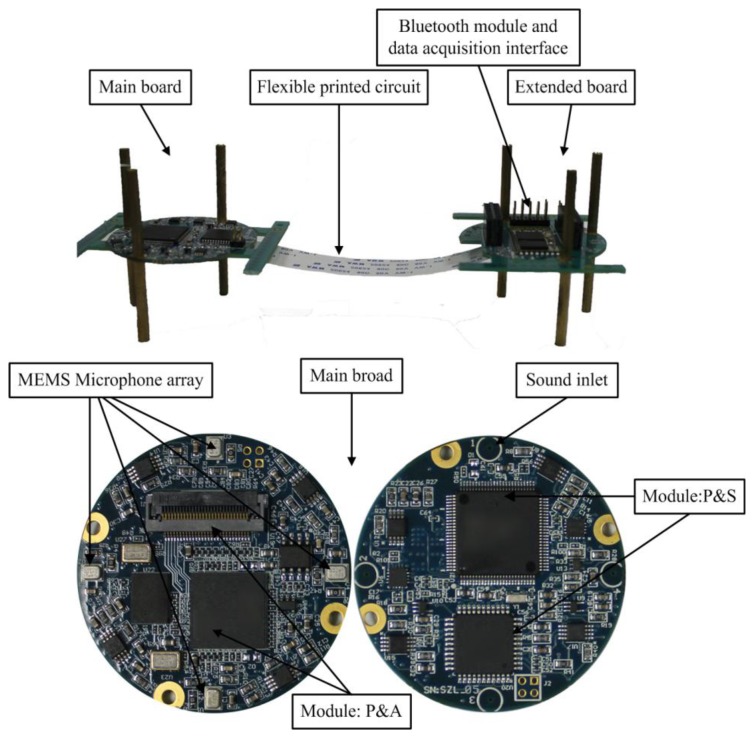
Photograph of the MEMS microphone array system, array aperture is 4 cm.

**Figure 4.**
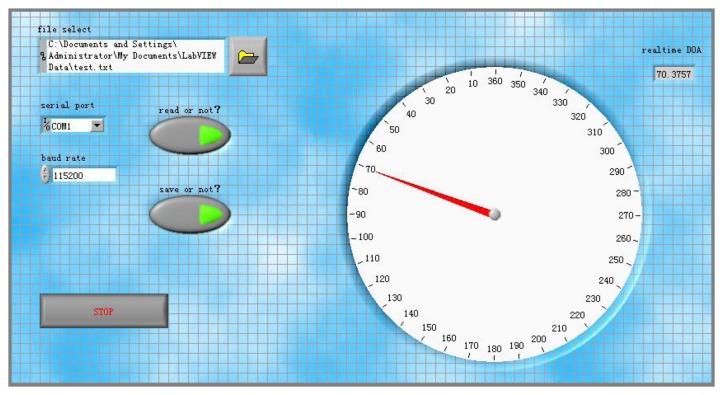
User interface of real-time DOA by UART.

As shown in Figure 3, the system consists of a main board and an extended board connecting by a flexible printed circuit (FPC). The main board consists of a uniform circular array system with four ADMP504 MEMS microphones (Analog Devices, Norwood, MA, USA), a ADSP21375 (Analog Devices, Norwood, MA, USA) as the core processor, MAXIM MAX11043, 4-Channel, 16-Bit, Simultaneous-Sampling ADCs (Maxim Integrated Products, Sunnyvale, CA, USA) and supplemental hardware circuits. The MAX11043 contains a versatile filter block and programmable-gain amplifier (PGA) per channel. The extended board contains a CSR BC6415 Bluetooth module (Cambridge Silicon Radio, Cambridge, UK), a data acquisition interface and debug interface. Figure 3 illustrates the hardware components that make up the system. Figure 4 shows the PC user interface of real-time DOA by UART in a LabVIEW 8.5 programming environment.

## 3. Signal Processing and Software Design

We first establish here the notation used before describing the direction finding method.

(1) The text in **bold** denotes vectors
(2) *E[X]* denotes the expectation of *X*
(3) *f* denotes the frequency domain, *ω* = 2*πf*
(4) Let *M* be the number of microphones in the array
(5) Let *L* be the length of samples
(6) Let *K* be the segment length of spatial coherence
(7) Let *N* be the scale of peak search

**Figure 5.**
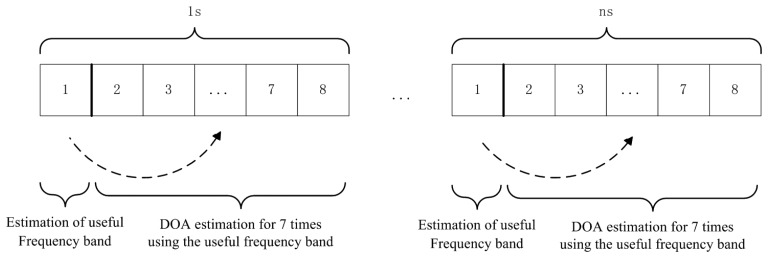
A schematic diagram of direction finding method.

The sampling rate of the system is 8,192 Hz. To ensure the accuracy of spatial coherence and direction finding, 1,024 samples (1/8 s) are used for calculating the spatial coherence and DOA estimation. One second is divided into two parts. As shown in Figure 5, the first 1/8 s in one second is used to estimate the useful frequency band for direction finding and seven DOA estimations are generated during the last the last 7/8 s using the frequency band.

### 3.1. Spatial Coherence

Wind noise is the most common interference outdoors. The wind turbulence on the microphone is comparatively incoherent and its speed is much slower than that of sound [23]. Two conclusions can be drawn as follows.

- The wind noise occupies a relatively lower frequency band compared to the vehicle sound
- Coherence can serve as a criterion to separate the wind noise and the vehicle bands

Spatial coherence is a similarity indicator for signals in the frequency domain. It describes the coherence between two measures at two locations [21]. Coherence function via overlapped Fourier transform is given by Equation (1), where *X* and *Y* are the frequency domain representations of the signals *x* and *y*:

(1) $$\gamma_{xy}(f)=\frac{\langle {\text{X}}^{*}Y\rangle }{\sqrt{\langle {\text{X}}^{*}X\rangle \cdot \langle Y^{*}Y\rangle }}$$

Taking FFT time duration *T*, and time delay *D* into consideration, an analytical estimation of the bias *E*[*γ̂*] is given as a function of the true coherence *γ* [24]:

(2) $$E\left[\hat{\gamma }\right]-\gamma ≅-\frac{-2\left|D\right|}{T}\gamma +{\left(\frac{\left|D\right|}{T}\right)}^{2}\gamma$$

In our case, T = 1/8 s (1024 samples), D = 8.31 × 10^−5^ s (array aperture of 4 cm):

$$E\left[\hat{\gamma }\right]-\gamma \approx 10^{-3}$$

**Figure 6.**
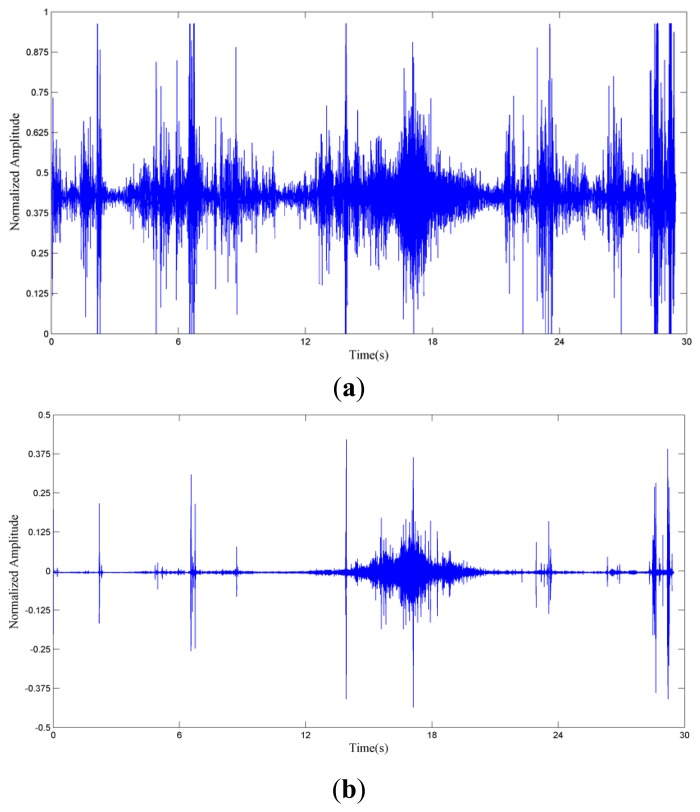

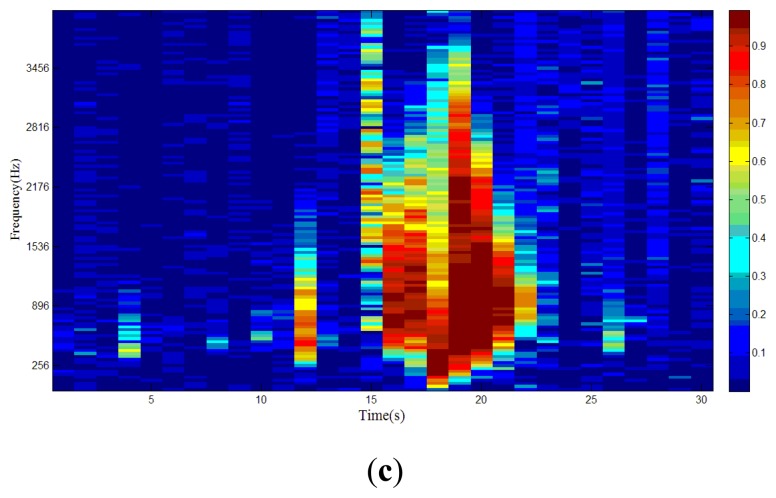
(**a**) Acoustic signal of a car passing the microphone array in field; (**b**) Filtered signal of (**a**) with a high-pass filter; (**c**). Spatial coherence of (**a**).

We use 1/8 s in one second to estimate the spatial coherence of the frequency band. *γ_xy_*(*f*) describes the coherence between two measures at two locations. The first step of the method is to test the spatial coherence for each frequency bin in the first 1/8 s. The sound of a passing vehicle contributes a different power fraction to different frequency bins. To identify the useful frequency band of the signal, we check whether the spatial coherence is above the threshold in each frequency bin. In this paper, 0.7 is chosen by simulation and experiment. Figure 6a shows the acoustic signal of a car passing the microphone array and the wind scale [25] is 4. A high-pass filter is applied to the signal to remove the influence of wind in Figure 6b. The 3 dB cut-off frequency of the filter is 445 Hz.

The car passes the microphone array between 16 s and 22 s. Spatial coherence is depicted in Figure 6c to show whether the frequency bin is dominated by vehicle or wind noise. If the spatial coherence of certain frequency bin is larger than 0.7, then this bin will be used for direction finding, otherwise it will be discarded.

### 3.2. Directional Spectrum Estimation

The MUSIC estimator is used to compute a directional spectrum in this paper. In some application, the acoustic signal of vehicle is considered as wideband. However, when the microphone array is small, the sound of a vehicle could be viewed as a narrowband signal [26]. Comparing with reference [18], some approximation of the MUSIC algorithm should be presented for vehicle DOA estimation in this paper.

The MUSIC algorithm is based on the fact that the array manifold ***a***(***θ***, *ω*_0_) and the noise eigenvectors ***E****_N_* are orthogonal to each other. Wideband MUSIC algorithms for acoustic sources focus on the fact that the array manifold changes as the frequency varies and hence one either has to calculate all the frequencies separately (incoherent wideband MUSIC) or find a focusing matrix and transform all the frequencies into a single one (coherent wideband MUSIC). However, the two methods will greatly increase the computational load, and therefore, they are not suitable for portable real-time applications, for example UGS, whereas the power supply is limited.

The array manifold changes as the frequency varies, while the decrease of the array aperture will make the change of array manifold smaller. In other words, the error caused by frequency dispersion declines as the array aperture becomes smaller. In this paper, as the aperture of the array is as small as 4 cm and the acoustic signal of vehicle is limited, the error of DOA estimation caused by frequency change in array manifold is negligible. With spatial coherence limiting the signal band, we use *ω*_0_ = 2π*(*f_L_* + *f_H_* / 2 for the band of direction finding. The overall direction finding method is now presented in a step-by-step format:

In slot 1 of Figure 5:

STEP-0 Calculate the spatial coherence of signals from the first two microphones, and choose the useful frequency band of [*f_L_*, *f_H_*] using the threshold of 0.7.

In slot 2–8 of Figure 5:

STEP-1 Collect L (1024) samples of data from the small aperture array of M sensors (4 microphones).
STEP-2 Calculate the Fourier transforms ***X*** of the signals of different microphones.
STEP-3 Construct the covariance matrices ***S*** corresponding to ***S*** = XX* using the frequency band [*f_L_*, *f_H_*] estimated from spatial coherence.
STEP-4 ***E****_N_* and ***a***(***θ***, *ω*_0_) (*ω*_0_ = 2π* (*f_L_* + *f_H_*)/2) are the noise subspace and the array manifold in reference [18]. Isolate the source locations as the maxima of the pseudo-spectrum *P_MUSIC_*(***θ***) = [‖a*(θ, *ω*_0_***)E****_N_*‖^2^]^−1^

**Figure 7.**
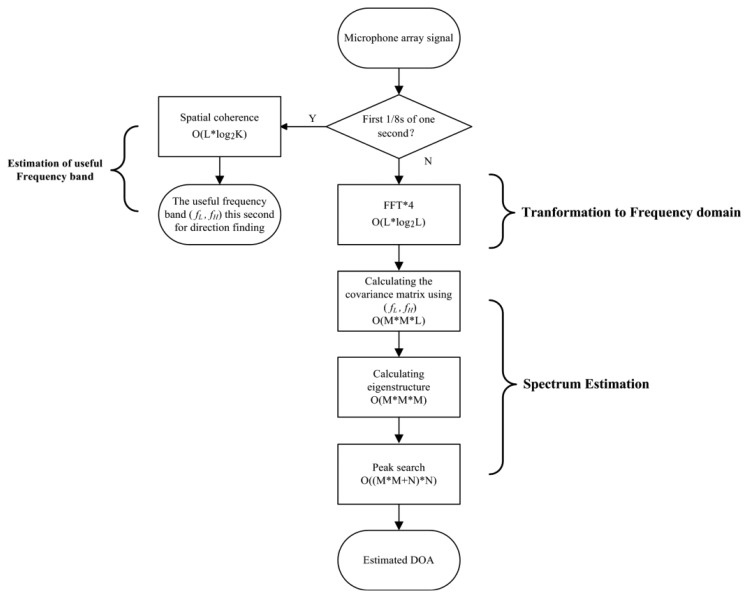
Flowchart of the moving vehicle direction finding method and time complexity.

Our method differs from the narrowband MUSIC algorithm [18] in STEP-3 and STEP-4. We spread the signal band to [*f_L_*, *f_H_*] in STEP-3, and in STEP-4, we use *ω*_0_ = 2π* (*f_L_* + *f_H_*)/2 for the array manifold in the direction finding frequency band. Compared to the wideband MUSIC algorithm, the complexity of our approximation is greatly reduced. Experiments in Section 4 show that the approximations will not cause performance degradation because the band-limited acoustic signal of vehicle can be considered as a narrowband source as the aperture of the array is very small. The proposed method is applied in a SHARC DSP in the system, equipped with a 75 MHz clock. The total time elapsed is 38 ms for 1,024 samples, with a sampling rate of 8,192 Hz. The computational complexity of the proposed method is shown in Table 2 and Figure 7.

## 4. System Verification and Experimental Results

**Figure 8.**
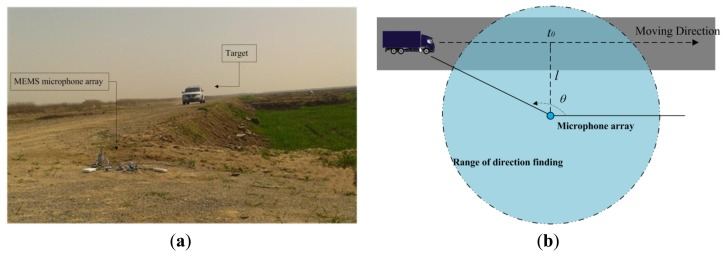
(**a**) Photograph of the experimental environment; (**b**) A schematic diagram of the direction finding experiment.

**Figure 9.**
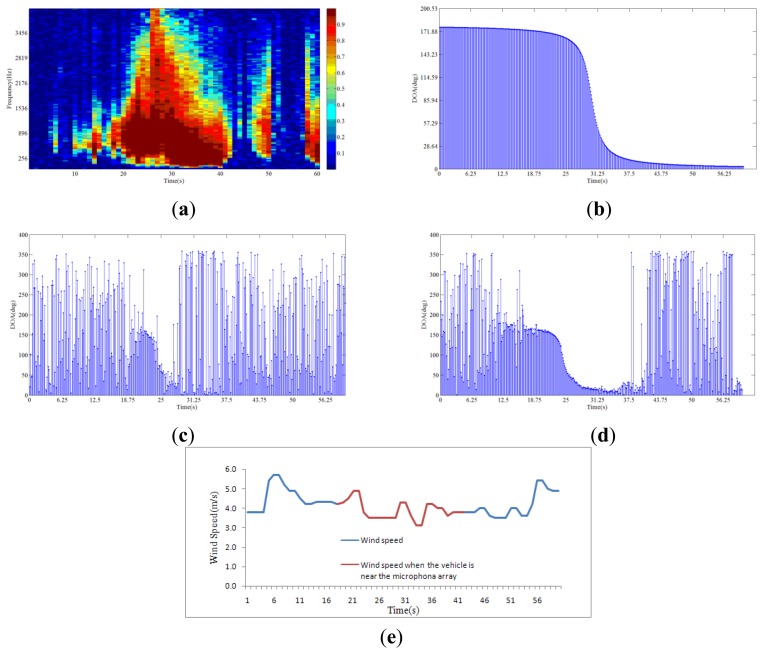
(**a**) Spatial coherence of the array signal in 1 minute; (**b**) ideal DOA of a car passing a microphone array; (**c**) DOA estimation with MUSIC algorithm using the entire frequency band; (**d**) DOA estimation using the method in this paper; (**e**) The recorded wind speed.

Experimental studies were performed from June 2012 to December 2013 on Chongming Island, Zhoushan Island (the third and fourth biggest islands in China) and a suburban district around Shanghai to demonstrate the feasibility of the system and the direction finding method proposed in this paper in the field. In Figure 8a, a car (a Dodge SUV) is passing the MEMS microphone array system. As shown in Figure 8b, assuming that the velocity of the vehicle is uniform, the DOA of the car satisfies the inverse tangent law of Figure 9b:

$$\theta =\frac{\pi }{2}+\text{arctan}(\frac{v(t_{0}-t)}{l}),t\in R$$

Figure 9a shows the spatial coherence of the array signal. In Figure 9c, the entire frequency band is used for direction finding, including the low frequency bin. In Figure 9d, the frequency bin with spatial coherence bigger than 0.7 is used so that the low frequency wind noise is discarded. It is shown in Figure 9c and Figure 9d that using the spatial coherence as a threshold to limit the processing signal band will improve the direction finding performance. The recorded wind speed at the time is shown in Figure 9e.

Different kinds of vehicles are used as targets for direction finding. Sorting the vehicles by ascending sound pressure level (SPL), the order is as follows: electric bicycle, car, bus, truck, tracked vehicle. The UGS works under different weather conditions within its area of operation, therefore the wind scale and range of direction finding is provided. The SPL, wind scale and range of direction finding reflect the signal to noise ratio (SNR). As for each target, the maximum wind level in the test and range of direction finding are different. For the relative conditions of different vehicles, the noise is a minimum 5 dB lower than the emitter. The estimation error of DOA is the RMSE from the fit of the inverse tangent within direction finding range. The experimental results show that the system could determine the DOA of different vehicles in the presence of wind and the accuracy is within 6° in relative range and wind scale. Table 3 lists the results of the experiments.

In Table 4, different designs and performances of six systems are listed. In Table 5, we compare our method in terms of computational complexity with time delay estimation (TDE) method, incoherent wideband MUSIC (IWM), coherent wideband MUSIC (CWM), and maximum likelihood (ML) method. The number of samples used for direction finding is 1,024. The sampling rate is 8,192 Hz. All of the methods are executed in the Matlab 2008a environment on a personal computer (dual core, 2.9 GHz-frequency processor and 2 GB memory).

In general, the aperture of our system is very small (4 cm) which is an advantage for portability and mobility, but a challenge for high accuracy direction finding. Compared with other systems, our system design has a moderate sampling rate and computational complexity. In systems No. 1–3 (Table 4), TDE exceeds our method in computational complexity, however the accuracy is low and it features a high sampling rate. Concerning IWM and CWM, the accuracies are close, yet the computational complexity of our method is much lower. ML has high accuracy but the computational complexity is too high for real-time processing. Moreover, while most of the systems mentioned the problem of wind noise, none of them have actually proposed a solution dealing with it and their experimental environment is low wind. Based on our experiments, the spatial coherence method enhances the performance of direction finding in the presence of wind noise. The experimental results and comparisons with other systems confirm the excellent comprehensive performance of the proposed system.

## 5. Conclusions

In this paper, a real-time direction finding system is implemented based on a SHARC DSP processor. An approximation of the narrowband MUSIC algorithm is applied in the system for its advantages of accuracy and relatively low complexity for a small aperture array. By means of spatial coherence, the influence of wind noise is greatly reduced and the direction finding performance is enhanced. Experiments at different locations have demonstrated that the system is able to locate different types of vehicles with an accuracy of 6°. The system is mainly designed for vehicle direction finding using a UGS system. However, the system could also provide a reference for other applications such as video conferencing and speaker tracking.

## Figures and Tables

**Table 1.** The root-mean-square error (RMSE) of direction finding using MUSIC algorithm for different microphone arrays.

**Array Aperture (cm)**	**RMSE of Direction Finding**						

	**20 dB**			**10 dB**			**Room Music**

	**500 Hz**	**1000 Hz**	**2000 Hz**	**500 Hz**	**1000 Hz**	**2000 Hz**	
Four microphones							
0.5	8.33	4.05	1.89	35.12	13.06	5.94	16.75
2	2.01	1.03	0.50	6.16	3.23	1.57	3.25
4	1.03	0.50	0.25	3.16	1.53	0.80	2.25
Three microphones							
0.5	9.14	4.48	2.23	38.67	15.33	7.07	30.50
2	2.25	1.15	0.56	7.29	3.44	1.82	15.46
4	1.15	0.54	0.28	3.60	1.81	0.93	8.46

**Table 2.** Computational complexity of the proposed method.

**Spatial Coherence**		**Direction Finding by the MUSIC Algorithm**			
Time	7.2 ms	38.0 ms			

		FFT	Calculation of the covariance matrix	Calculation of eigenstructure a	Peak search
Percentage of each part		15.6%	6.6%	10.2%	67.6%

a The covariance matrix in STEP-3 is a Hermitian Matrix and the method of Jacobi for the eigen-decomposition algorithm of Hermitian matrix is used in this method [27].

**Table 3.** Experimental results.

**Target**	**Maximum Wind Scale in Test**	**Test Times**	**Estimation Error of DOA within Range (deg)**	**Range (m^2^)**
Electric bicycle	3	≥20	5.82	5,000
Car	4	≥40	3.10	10,000
Bus	4	≥40	2.20	20,000
Truck	4	≥40	2.61	20,000
Tracked vehicle	5	≥40	2.74	50,000

**Table 4.** Performance comparison with other systems.

**No.**	**Target**	**Aperture (cm)**	**Accuracy (deg)**	**Array**	**Number of Microphones**	**Method**	**Sampling Rate (kHz)**
1	Car [4]	15	5	ULA	4	TDE	44.1
2	Trailer [5]	20	20	ULA	3	TDE	48
3	Motor vehicle [6]	102	12	ULA	7	TDE	10
4	Tracked vehicle [1]	>100 ^a^	1.5	UCA	5	IWM	8.192
5	Tank [28,29]	20.23	<2	UCA	12	CWM	N/A
6	AAV ^b^[7]	N/A ^c^	High	Random	N/A	ML	4.96

a The aperture of the microphone array is not given, it is estimated from the picture in [1];

b AAV means amphibious assault vehicle;

c N/A means not available.

**Table 5.** Time elapsed for different methods.

**Methods**	**TDE**	**IWM**	**CWM**	**ML**	**Ours**
Time elapsed (s)	0.0022	0.1020	0.0724	0.3114	0.0074
